# Lipid recognition propensities of amino acids in membrane proteins from atomic resolution data

**DOI:** 10.1186/2046-1682-4-21

**Published:** 2011-12-14

**Authors:** Mizuki Morita, AVSK Mohan Katta, Shandar Ahmad, Takaharu Mori, Yuji Sugita, Kenji Mizuguchi

**Affiliations:** 1 Department of Fundamental Research, National Institute of Biomedical Innovation, 7-6-8 Saito Asagi, Ibaraki, Osaka, Japan; 2 Institute of Bioinformatics Research and Development, Japan Science and Technology Agency, 5-3 Yonbancho, Chiyoda-ku, Tokyo, Japan; 3 RIKEN Quantitative Biology Center, 7-1-26 Minatojima-minamimachi, Chuo-ku, Kobe, Hyogo, Japan; 4 RIKEN Advanced Science Institute, 2-1 Hirosawa, Wako-shi, Saitama, Japan; 5 RIKEN Advanced Institute for Computational Science, 7-1-26 Minatojima-minamimachi, Chuo-ku, Kobe, Hyogo, Japan

## Abstract

**Background:**

Protein-lipid interactions play essential roles in the conformational stability and biological functions of membrane proteins. However, few of the previous computational studies have taken into account the atomic details of protein-lipid interactions explicitly.

**Results:**

To gain an insight into the molecular mechanisms of the recognition of lipid molecules by membrane proteins, we investigated amino acid propensities in membrane proteins for interacting with the head and tail groups of lipid molecules. We observed a common pattern of lipid tail-amino acid interactions in two different data sources, crystal structures and molecular dynamics simulations. These interactions are largely explained by general lipophilicity, whereas the preferences for lipid head groups vary among individual proteins. We also found that membrane and water-soluble proteins utilize essentially an identical set of amino acids for interacting with lipid head and tail groups.

**Conclusions:**

We showed that the lipophilicity of amino acid residues determines the amino acid preferences for lipid tail groups in both membrane and water-soluble proteins, suggesting that tightly-bound lipid molecules and lipids in the annular shell interact with membrane proteins in a similar manner. In contrast, interactions between lipid head groups and amino acids showed a more variable pattern, apparently constrained by each protein's specific molecular function.

## Background

About 20-30% of all proteins encoded in a typical genome are estimated to be localized in membranes [1,2], where protein-lipid interactions play crucial roles in the conformational stability and biological functions of membrane proteins. Many experimental studies have suggested that physico-chemical properties of the membrane lipid bilayer influence the stability and function of membrane proteins. The thermal [3,4] and chemical [5] stability of the potassium channel KcsA has been shown to vary according to the lipid composition of the membrane bilayer. It has also been shown that the lipid composition affects protein functions including: ion transport in KcsA [6,7] and the Ca^2+^-ATPase of sarcoplasmic reticulum [8,9], phosphorylation by the diacylglycerol kinase [10] and chemical compound transport by the mechanosensitive channel of large conductance MscL [11]. To complement these experimental studies, statistical analyses have been carried out to reveal amino acid preferences and conservation patterns within the lipid bilayer environment [12-16] using available sequence and structural data. The patterns emerging from these statistical analyses should reflect implicitly the effects of lipid molecules on the structural formation and stability of membrane proteins. However, few of the previous computational studies have taken into account the atomic details of protein-lipid interactions explicitly. A notable exception is all-atom molecular dynamics (MD) simulations; it has become possible to apply the technique to membrane proteins in conditions mimicking biological membranes (reviewed recently by Khalili-Araghi and co-authors [17]). All-atom MD simulations enable us to inspect protein-lipid interactions in atomic details [18,19] and can reveal the role of lipids in protein function [20], albeit for a small selection of specific lipid and protein molecules.

In this paper, we attempt to understand the nature of protein-lipid interactions using a computational approach. Given the limited number of crystal structures containing lipid molecules, we decided to combine all known biological phospholipids together and classify the atomic interactions into those involving the "head" and "tail" parts of the lipids. The head and tail groups can be found in most phospholipids constituting a biological membrane and define one of the most essential chemical features of these molecules. Thus, we ask more specifically: "How are the head and tail portions of lipid molecules recognized by amino acid residues in membrane proteins?"

To answer this question, we utilized two available data sources, crystal structures and MD trajectories. Using the crystal structure data, we can include and examine various kinds of proteins and lipids, although the number of lipid molecules observed in each solved structure is limited. Using the MD data, we can obtain detailed information about all the lipid molecules surrounding a protein, although such an analysis is possible only for a small set of protein and lipid types. The combination of these two data sources allows us to assess the biases resulting from a limited variety of data in each data source. The results revealed a common pattern of lipid tail-amino acid interactions observed in both the crystal structures and MD trajectories. We show that the recognition of lipid tails can be explained largely by general lipophilicity and that this effect dominates in the two different situations represented by the crystal structure and MD datasets. In contrast, lipid head groups showed a more complicated and diverse pattern and we discuss how our observations can be related to known experimental data and previously proposed concepts concerning protein-lipid interactions.

## Methods

### Lipid definition and dataset

Lipids in this paper were defined as phosphoglycerides that consisted of one or two fatty acids linked through glycerol phosphate to zero or one polar group, and their mimetic compounds. First, an initial list of three-letter HET IDs of lipids in the Protein Data Bank (PDB) [21] was obtained by keyword searches against the Chemical Component Dictionary (CCD) through Ligand Expo [22] and PDBeChem [23] using all the MeSH terms below 'Glycerophosphates' in the MeSH hierarchy. Next, mimetic compounds were found by the "Similar Compound Search" function at PubChem [24] and RCSB PDB [25]. Finally, all the collected compounds were manually checked to determine whether they met the definition of lipids above. A total of 98 HET IDs were collected (Table 1) and used to search for proteins in contact with lipids in the PDB repository (see the next section).

**Table 1:** List of HET IDs for the phospholipids considered in this paper


2DP	3PE	3PH	3PI	4PT	6PH	6PL	7PH	8PE	9PE
AGA	B7N	CDL	CDN	CN3	CN5	CN6	CPL	DGG	DLP
DPG	DR9	EPH	GP7	HGP	HGX	HHG	HI5	IP9	L1P
L3P	L4P	L9Q	L9R	LAP	LHG	LIO	LOP	LP3	LPC
LPE	LPP	LPS	LPX	MC3	MYY	NKN	NKO	NKP	NKQ
NKR	OPC	OZ2	P0E	P3A	P42	P6L	PA6	PBU	PC1
PC2	PC6	PC7	PC9	PCF	PCK	PCW	PD7	PDK	PEE
PEF	PEH	PEK	PEV	PEW	PFS	PGK	PGM	PGT	PGV
PGW	PIB	PIE	PIF	PII	PIO	PLC	PLD	PLX	POV
PS2	PS6	PSC	PSF	PT5	PTY	PX4	XPX		

### Crystal structure data of protein-lipid complexes

Using the HET IDs listed in Table 1, a local repository of the PDB (updated on February 9, 2011) was scanned for the crystal structures of proteins that contained these lipid molecules. Retaining only those structures solved at 4.0 Å resolution or better (ignoring structures solved by NMR and other methods, for which resolution was unavailable), a total of 290 protein-lipid complexes were obtained initially, consisting of 1,657 chains. Protein chains that were smaller than 30 residues, that contained one or more non-standard amino acid residues (except for selenomethionine, which was treated as MET) and that had no lipid contacts (see below for the definition of contacts) were removed from this set, leaving 1,497 protein chains. These sequences were clustered using the BLASTClust program (available from the BLAST [26] distribution) at a 25% sequence identity cutoff, resulting in 148 clusters. Clusters in which all the members had less than five residues in contact with lipids were discarded. The remaining clusters were classified into transmembrane (TM) and non-transmembrane (non-TM) in the following manner. A cluster was initially annotated as either TM, if any of its members was found in the PDBTM [27] or OPM databases [28] (both downloaded on February 6, 2011), or non-TM otherwise. To confirm the presence (or absence) of TM helices, PDB2TMD [29] was run, followed by manual inspection to ensure that all the proteins were correctly annotated as TM or non-TM. From each cluster, the protein chain with the highest number of lipid-contacting residues was selected as the representative, producing 45 TM and 27 non-TM protein chains (Table 2).

**Table 2:** List of transmembrane (TM) and non-transmembrane (non-TM) protein chains in complex with lipids

Proteincode^a^	Protein name	Lipid code^b^	Total contacts^c^	Chainlength
**(a) Transmembrane (TM) protein chains**				
1gzm_A	Rhodopsin	PEF	5	329
1kqg_B	Formate dehydrogenase-N (Fdn-N)	CDL	7	289
1kqg_C	Formate dehydrogenase-N (Fdn-N)	CDL	5	216
1m56_D	Cytochrome c oxidase	PEH	16	42
1nen_C	Succinate dehydrogenase (SQR)	CDN, EPH	19	129
1nen_D	Succinate dehydrogenase (SQR)	CDN	9	113
1pp9_Q	Cytochrome bc1 complex	CDL, PEE	15	241
1pp9_T	Cytochrome bc1 complex	CDL, PEE	12	76
1vf5_D	Cytochrome b6f complex	OPC	5	168
1vf5_N	Cytochrome b6f complex	OPC	6	202
1x0i_1	Bacteriorhodopsin (BR)	L3P	14	215
1xio_A	Sensory rhodopsin (SR)	PEE	32	217
1zoy_D	Succinate:ubiquinone oxidoreductase (SQR)	EPH	10	102
2b6o_A	Aquaporin-0 (AQP0)	MC3	27	235
2bl2_I	Vacuolar-type (V-type) sodium ion-pumping adenosine triphosphatase (Na^+^-ATPase)	LHG	14	156
2brd_A	Bacteriorhodopsin (BR)	DPG	47	222
2c3e_A	ADP/ATP translocase 1	CDL	40	293
2e75_B	Cytochrome b6f complex	OPC	13	160
2e76_F	Cytochrome b6f complex	OPC	5	32
2eau_A	Ca^2+^-ATPase	PTY	19	994
2eim_W	Cytochrome c oxidase	CDL	5	58
2ein_O	Cytochrome c oxidase	CDL, PEK, PSC	18	226
2h89_C	Succinate:ubiquinone oxidoreductase (SQR)	PEE	5	139
2hg3_H	Reaction center	CDL, PC9	14	240
2hh1_L	Reaction center	CDL, PC7, PC9	10	281
2hhk_M	Reaction center	CDL, PGK, PGT	23	302
2irv_B	Rhomboid protease (GlpG)	PGV	11	179
2r9r_B	Voltage-dependent K^+ ^(Kv) channel	PGW	35	386
2wll_B	Potassium Channel (Kir)	PLC	6	266
2z73_B	Rhodopsin	PC1	5	347
3a7k_A	Halorhodopsin (HR)	L1P, L3P	33	259
3abl_N	Cytochrome c oxidase	CDL, PEK, PGV, PSC	29	513
3abl_P	Cytochrome c oxidase	CDL, PEK, PGV	85	259
3abm_G	Cytochrome c oxidase	CDL, PEK, PGV	25	83
3ag4_Z	Cytochrome c oxidase	PGV	5	43
3bz2_A	Photosystem II (PSII)	LHG	6	335
3bz2_C	Photosystem II (PSII)	LHG	5	447
3bz2_D	Photosystem II (PSII)	LHG	6	340
3cx5_C	Cytochrome bc1 complex	6PH, 7PH, 8PE, 9PE, CN3, CN5	42	385
3ddl_B	Xanthorhodopsin (XR)	PCW, PX4	6	250
3eam_C	Bacterial ligand-gated ion channel homologue (GLIC)	PC1	23	311
3egw_C	Nitrate Reductase A (NarGHI)	AGA	10	224
3emn_X	Voltage-dependent anion channel (VDAC) 1	MC3	7	283
3h1j_R	Cytochrome bc1 complex	PEE, PLC	11	196
3h1j_W	Cytochrome bc1 complex	PEE, PLC	9	59
				
**(b) Non-transmembrane (non-TM) protein chains**				
1bp1_A	Bactericidal/permeability-increasing protein (BPI)	PC1	47	456
1bwo_A	Nonspecific lipid transfer protein (ns-LTP1)	LPC	20	90
1cqx_B	Flavohemoglobin	DGG	15	403
1l8s_B	Phospholipase A2 (PLA2)	LPE	11	124
1lsh_A	Lipovitellin (LV-1N, LV-1C)	PLD	53	954
1lsh_B	Lipovitellin (LV-2)	PLD	9	174
1s9a_B	4-chlorocatechol 1	HGP	13	256
1tuk_A	Type 2 nonspecific lipid transfer protein (ns-LTP)	PGM	11	67
1un8_A	Dihydroxyacetone kinase	MYY	15	542
1y9t_A	Lipoprotein MxiM	HHG	7	110
1yuc_B	Nuclear receptor liver receptor homolog 1 (LRH-1)	EPH	22	240
2azq_A	Catechol 1	PCF	9	309
2e2x_B	Sec14 homology module of neurofibromin	PEV	22	250
2obd_A	Cholesteryl ester transfer protein (CETP)	PCW	41	472
2qgu_A	Phospholipid-binding protein	PEF	12	179
2rak_A	PX-BAR membrane-remodeling unit of sorting nexin 9 (SNX9)	PIB	5	382
2rkn_A	Defective in induced resistance 1 protein (DIR1)	LP3	22	77
2vwa_B	Soluble domain of up-regulated in infective sporozoites 3 (UIS3)	PTY	12	100
2z0p_D	PH domain of Bruton's tyrosine kinase	4PT	13	161
2ze9_A	Phospholipase D	PD7	10	504
3a7c_A	Extracellular domain of Toll-like receptor 2 (TLR2)	PDK	13	549
3bib_X	T cell immunoglobulin mucin protein 4 (TIM-4)	PSF	9	109
3cx9_A	Human serum albumin (HSA)	LPX	17	582
3e3c_B	Global regulator of LEE repressor (GrlR)	HHG	15	118
3k7t_A	6-hydroxy-L-nicotine oxidase	GP7	17	425
3mdb_C	Arf-GAP with dual PH domain-containing protein 1	IP9	9	365
3mtx_B	Myeloid differentiation factor 1 (MD-1)	PGT	18	140

^a ^Protein ID and chain ID.

^b ^HET IDs of all contacting lipids in the complex.

^c ^Total number of residues with lipid contacts.

Although the resolution cutoff for data collection has been set to 4.0 Å, the worst resolution of any included structure was 3.7 Å. Also, only two protein chains in the TM data set had worse than 3.5 Å resolution, and only four had worse than 3.0 Å resolution. All the non-TM structures had 3.0 Å or better resolution. Thus, the final list contained most proteins solved at a decent resolution. All the statistical analyses in this paper were based on these protein chains unless otherwise specified. Although no conscious selection was made, the protein chains in the TM dataset were mostly helical, with the only exception of a beta barrel anion channel protein (PDB:3emn).

### MD simulation data

MD simulations were carried out for three TM proteins, the protein-conducting channel *Thermus thermophilus *SecYE (ttSecYE) [30], Ca^2+^-ATPase of skeltal muscle sarcoplasmic reticulum [31] and *Methanococcus jannaschii *SecYEβ (mjSecYEβ) [32], with the membrane lipids POPC (palmitoyl-oleyl-phosophatidylcholine) (for ttSecYE and mjSecYEβ) and DOPC (dioleyl-phosphatidylcholine) (for Ca^2+^-ATPase), respectively. MD trajectory data were obtained from the all-atom model simulations of these proteins in the fully hydrated lipid bilayer using the isothermal-isobaric ensemble (NPT) and constant area isothermal-isobaric ensemble (NPAT) [20,30,33]. The total simulation length was 100 ns for each simulation run. A total of 1,000 snapshots taken every 100 ps were used for the analysis.

### Amino acid-lipid contacts and propensity scores

Various types of amino acid-lipid contacts exist in protein-lipid complexes. They were broadly grouped into (1) hydrogen-bonded, (2) van der Waals and (3) salt bridges. These contacts were defined by using the HBPLUS program [34] with the standard atomic radii from the PDB het dictionary [35]. The default definitions of van der Waals interactions and hydrogen bonds were used to identify the amino acid-lipid contacts. According to the algorithm used in HBPLUS, hydrogen atoms were first added to the protein structure and then a hydrogen bond was identified if (i) the donor-acceptor distance was less than 3.9 Å, (ii) the hydrogen-acceptor distance was 2.5 Å and (iii) all three angles D-H-A, D-A-AA and H-A-AA were greater than 90°. (D, A, H and AA stands for donor, acceptor, hydrogen, and acceptor antecedents, respectively.) For aromatic interactions, the angles D-A-AX and H-A-AX (for amino-aromatic interactions) were also required to be less than 20°. (Further details and a list of acceptor and donor atoms can be found at [36].) The amino acid residue-lipid contacts were further classified into lipid tail and head group contacts. Specifically, the tail group of a lipid was defined as the set of all the atoms from the aliphatic tail to the carbon atom next to the carbonyl group of the fatty acid (or the corresponding carbon atom in a mimetic lipid). The head group of a lipid was defined as all the other atoms. The tail groups are predominantly hydrophobic, while the head groups are hydrophilic.

All contact preferences were measured in terms of a propensity score. First, a propensity score for each of the 20 amino acid residues was computed for each protein. The propensity *P_i _*of residue type *i *(e.g., LYS; *i *= 1 ... 20) in a protein was defined as the relative number of residues of type *i *in contact with lipids, normalized by the overall relative number of residues in contact with lipids:

(1) $$P_{i}=\frac{N_{i}^{b}\;/)\;N_{i}}{N^{b}\;/)\;N}$$

where *N_i_^b ^*is the number of lipid binding amino acid residues of type *i*, *N_i _*is the total number of amino acids of type *i*, *N^b ^*is the total number of lipid binding residues and *N *is the total number of amino acid residues. All the counts were made within the given protein sequence. The propensity values range between 0 and ∞. An amino acid propensity value of 1 indicates a neutral preference to binding lipids, while propensity values of <1 and >1 show a low and high preference, respectively. If a residue type was not represented in a protein chain, its propensity was undefined and excluded from further statistics. If a particular amino acid type was present in the chain but was not binding to lipids, its propensity was 0. Finally, the propensity scores thus computed for each protein chain were averaged over a set of proteins to draw comparison between one set (e.g., TM) and another (e.g., non-TM). The standard error of the mean was estimated as $s/)\sqrt{n},$ where *s *is the sample standard deviation and *n *is the sample size (i.e., the number of protein chains in the set considered, for which the propensity was defined).

We derived all the contact statistics from the entire protein chains including the residues in extra-membranous loops, because lipid-contacting residues were found both in the TM helices and loops and also, to make a natural comparison between the TM and non-TM proteins. Focusing only on the TM regions would not change the overall statistics, as most TM proteins considered had only short loops (with the exception of the MD trajectory data for Ca^2+^-ATPase, for which the large extra-membranous domain was excluded from the analysis).

### Chi-square test and statistical significance

To determine whether a particular amino acid is statistically significantly over- or under-represented in contact with lipid head or tail atoms, we pooled all the contact counts in the TM or non-TM dataset (considering only those proteins with at least six residues forming a given type of contacts). The expected number *E_i _*of lipid binding residues of type *i *in a given dataset was computed as

(2) $$E_{i}=\frac{N_{i}\cdot N^{b}}{N}$$

where *N_i_*, *N^b ^*and *N *were as above but obtained for the entire dataset. It was then compared with the observed number *O*_i _of lipid binding residues of type *i *by using a Chi-square statistic:

(3) $$\chi_{i}^{2}=\frac{{\left(O_{i}-E_{i}\right)}^{2}}{E_{i}}$$

The calculated ${\chi_{i}}^{2}$ values were converted to p-values using the standard Chi-square table with a single degree of freedom.

### Propensity in MD trajectories

To calculate propensity scores from the MD data, a contact was defined using a non-integer value equal to the fraction of the snapshots, in which the amino acid residue under consideration was in contact with any lipid molecule. More precisely, the total number *N_(k)_^b ^*of lipid binding counts for the *k*th amino acid residue in each MD trajectory was defined as

(4) $$N_{\left(k\right)}^{b}=\frac{\underset{t}{\sum }I_{\left(k\right)}^{b}\left(t\right)}{\underset{t}{\sum }1}$$

where *I_(k)_^b^*(*t*) is 1 if the *k*th amino acid residue was in contact with any lipid molecule in snapshot *t*, and 0 for no contact. For example, within a trajectory of 1,000 snapshots, if ARG90 is observed to be interacting with lipids in 300 snapshots, then *N^b^_(ARG90) _*is 0.3. The total number of lipid binding amino acid residues of type *i *(i.e., *N_i_^b ^*in Eq. 1) can be then obtained by summing up these quantities for all the ARG residues.

### Lipophilicity scales of amino acids

Comparisons were made between the lipid propensity scores of residues derived from the TM and MD datasets and the thermodynamic free energy of transferring amino acid residues from water to the interface of POPC bilayer and to octanol. The latter (called the lipophilicity scales in this paper) was taken from the data provided in White and Wimley's paper [37]. For the lipophilicity scales, we kept the protonation states of ARG and LYS positive, ASP and GLU negative and HIS neutral.

### Correlation between propensity values of two datasets

Comparisons between residue preferences were made using scatterplots and Pearson's correlation coefficient defined as

(5) $$C=\frac{n\sum X_{i}Y_{i}-\sum X_{i}\sum Y_{i}}{\sqrt{n\sum X_{i}^{2}-{\left(\sum X_{i}\right)}^{2}}\sqrt{n\sum Y_{i}^{2}-{(\sum Y_{i})}^{2}}}$$

where *X_i _*and *Y_i _*represent propensity (or lipophilicity) values of residue type *i *in two datasets being compared.

The jackknife estimate of the standard error of the correlation coefficient was obtained as:

(6) $$\sigma^{2}=\frac{N+1}{N}\overset{N}{\underset{i=1}{\sum }}{\left(C_{\left(-i\right)}-<C>\right)}^{2}$$

where *C*_(*-i*) _is the correlation coefficient calculated from data with the *i*th amino acid type removed and <*C*> is the mean of *N *(= 20) such values. The square root of the quantity in Eq. 6 was shown as the estimated standard error.

## Results

### Amino acid propensities from the crystal structure and MD datasets

**Figure 1:**
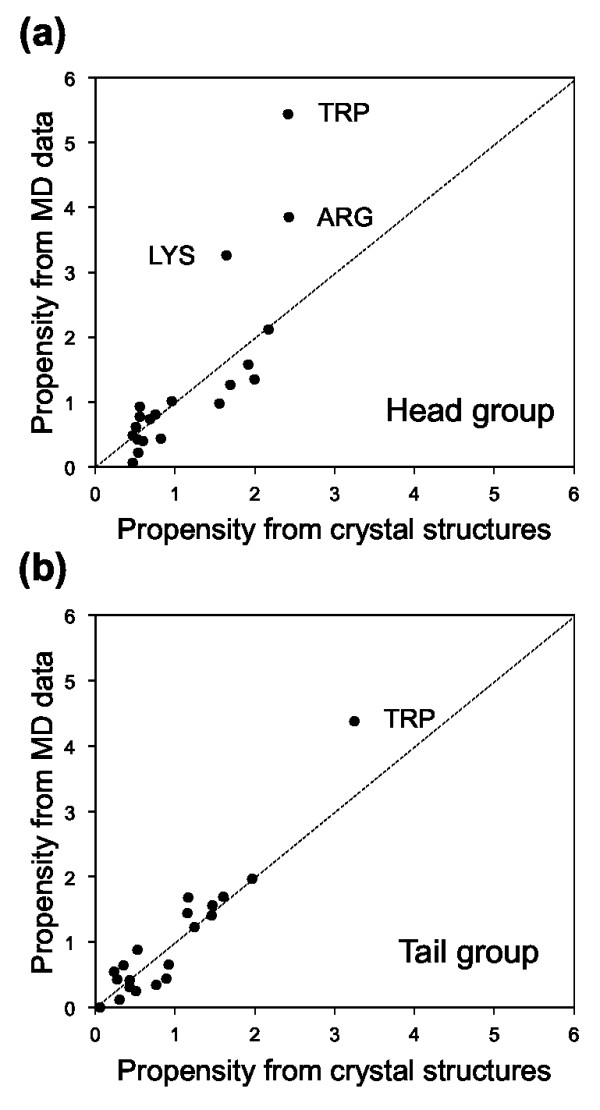
**Scatterplots of amino acid propensities for interacting with lipids derived from crystal structures and MD data**. For (a) lipid head and (b) tail groups. The correlation coefficients were 0.81 and 0.95 for the head and tail groups, respectively.

Amino acid propensities of membrane proteins contacting with lipid head and tail groups were derived from both crystal structures and MD simulations. Figure 1 shows scatterplots between the propensities from the crystal structure and MD datasets. The correlation coefficients between these two were 0.81 and 0.95 for the lipid head and tail group contacts, respectively (see also Tables 3 and 4). Although good agreements were observed in both the lipid head and tail group contacts, some points in the plot for the head group contacts do not lie close to a straight line (Figure 1a), especially when compared with the plot for the tail group contacts (Figure 1b). When the outliers in the head group plot (TRP, ARG, LYS) were removed, the correlation coefficient rose to 0.88, a value close to that of the tail group without TRP (0.90) (see also Additional file 1, Fig. S1).

**Table 3:** Amino acid propensities for interacting with lipids from crystal structures and MD trajectories, and lipophilicity scales

	Propensity from crystal structures^a^				Propensity from MD trajectories		Lipophilicity scale^b^	
			
	Head group		Tail group		Head group	Tail group	POPC	octanol
TRP	2.41	(1.04)	3.25	(0.63)	5.44	4.38	-1.85	-2.09
PHE	1.55	(0.50)	1.96	(0.43)	0.97	1.97	-1.13	-1.71
TYR	2.17	(0.57)	1.15	(0.26)	2.12	1.45	-0.94	-0.71
LEU	0.75	(0.17)	1.60	(0.23)	0.80	1.70	-0.56	-1.25
ILE	0.47	(0.09)	1.46	(0.27)	0.48	1.41	-0.31	-1.12
CYS	0.47	(0.32)	1.16	(0.93)	0.06	1.68	-0.24	-0.02
MET	0.96	(0.25)	1.47	(0.38)	1.01	1.56	-0.23	-0.67
GLY	0.53	(0.20)	0.43	(0.13)	0.42	0.42	0.01	1.15
VAL	0.60	(0.18)	1.24	(0.41)	0.40	1.23	0.07	-0.46
SER	0.68	(0.20)	0.89	(0.25)	0.73	0.44	0.13	0.46
THR	0.82	(0.25)	0.76	(0.15)	0.43	0.35	0.14	0.25
ALA	0.54	(0.15)	0.92	(0.19)	0.22	0.66	0.17	0.50
HIS	1.99	(0.39)	0.53	(0.09)	1.35	0.88	0.17	0.11
ASN	1.92	(0.47)	0.43	(0.13)	1.57	0.31	0.42	0.85
PRO	0.56	(0.37)	0.35	(0.12)	0.77	0.65	0.45	0.14
GLN	1.69	(0.98)	0.51	(0.26)	1.26	0.25	0.58	0.77
ARG	2.42	(0.65)	0.27	(0.21)	3.85	0.43	0.81	1.81
LYS	1.64	(0.58)	0.23	(0.09)	3.26	0.55	0.99	2.80
ASP	0.51	(0.32)	0.06	(0.04)	0.61	0.00	1.23	3.64
GLU	0.56	(0.57)	0.30	(0.13)	0.93	0.12	2.02	3.63

The amino acids are sorted in the ascending order of the lipophilicity scale for POPC interface.

^a ^Values in parentheses represent the estimated standard error of correlation. The average of the standard error is 0.41 for lipid head and 0.27 for tail groups.

^b ^The oxidation state of HIS has been taken as neutral. All ARG and LYS are taken as positively and all ASP and GLU are taken as negatively charged.

**Table 4:** Three-way relationships between the amino acid propensities for interacting with lipids from crystal structures and MD trajectories, and lipophilicity scales

		Propensity from crystal structures		Propensity from MD trajectories		Lipophilicity scale^a^	
				
		Head group	Tail group	Head group	Tail group	POPC	octanol
**Propensity from crystal structures**	**Head group**	1.00 (0.00)	0.19 (0.38)	0.81 (0.06)	0.32 (0.36)	-0.28 (0.27)	-0.16 (0.25)
	**Tail group**		1.00 (0.00)	0.33 (0.69)	0.95 (0.05)	-0.87 (0.07)	-0.82 (0.05)
							
**Propensity from MD trajectories**	**Head group**			1.00 (0.00)	0.49 (0.66)	-0.24 (0.49)	-0.06 (0.40)
	**Tail group**				1.00 (0.00)	-0.84 (0.05)	-0.75 (0.07)
							
**Lipophilicity scale**^a^	**POPC**					1.00 (0.00)	0.92 (0.03)
	**octanol**						1.00 (0.00)

All-against-all correlation coefficients between the properties presented in Table 3. Values in parentheses represent standard error in correlation (see Methods).

^a ^The oxidation state of HIS has been taken as neutral. All ARG and LYS are taken as positively and all ASP and GLU are taken as negatively charged.

The contact preferences for lipid head groups had larger variance among individual proteins than for tail groups (see Table 3 and the Discussion section below). Thus, two of the outliers, LYS and ARG, may be due to the small number of proteins in the MD dataset; ttSecYE had more ARG residues than the average in the crystal structure dataset [16], while mjSecYEβ had more LYS residues than the average. All these residues clustered in the membrane interfaces, especially on the cytoplasmic side. Such a bias would have resulted in the higher head propensities of LYS and ARG in the MD dataset, although further analysis is needed to confirm this notion. Particularly high propensities of TRP were observed in both scatterplots, suggesting that TRP residues are more frequently located in the regions that allow direct contacts with lipid molecules than in other regions (see Discussion below).

### Specific observations for each amino acid residue

Here, we describe the lipid head and tail group preferences of each amino acid residue observed in both the crystal structure and MD datasets (Table 3).

Only TRP and TYR were favored by both the lipid head and tail groups. These residues, with their amphiphilic nature, play a special role in the membrane-water interfaces. The small residues (GLY, SER, THR, ALA, PRO) were excluded from both lipid head and tail groups. Our previous study showed the propensities of the small residues on the protein surface in the TM region and around the membrane interfaces to be low, while those in the buried positions to be high [16]. These residues are thought to stabilize inter-helical contacts through non-conventional hydrogen bonds (Cα--H...O) [16,38]. The acidic residues (ASP, GLU), but not the basic ones (HIS, ARG, LYS), were also excluded from both lipid head and tail groups, consistent with the basic residues to occur favorably on the surface of the intracellular interface [16] (the positive-inside rule [39]).

For lipid head group contacts, hydrophilic residues, both basic (HIS, ARG, LYS) and uncharged polar (ASN, GLN), were favored, except for small (SER and THR) and acidic (ASP, GLU). TRP and TYR were the only hydrophobic residues favored by lipid head groups. For lipid tail group contacts, no hydrophilic residues were favored and all the hydrophobic residues (TRP, PHE, TYR, LEU, ILE, CYS, MET, VAL) were favored, except for small ones (PRO, ALA, GLY).

### Comparison with the lipophilicity scales

We then compared the amino acid propensities with the experimentally determined lipophilicity scales, which were derived from transfer free energies of model peptides from water to POPC membrane interface and to bulk octanol [37]. (The correlation coefficients were calculated by using the raw values of the amino acid propensities and the lipophilicity scales, as described in Methods.) The amino acid propensities and the lipophilicity scales are summarized in Table 3, and a comprehensive list of correlation coefficients between the three sets of values is shown in Table 4.

**Figure 2:**
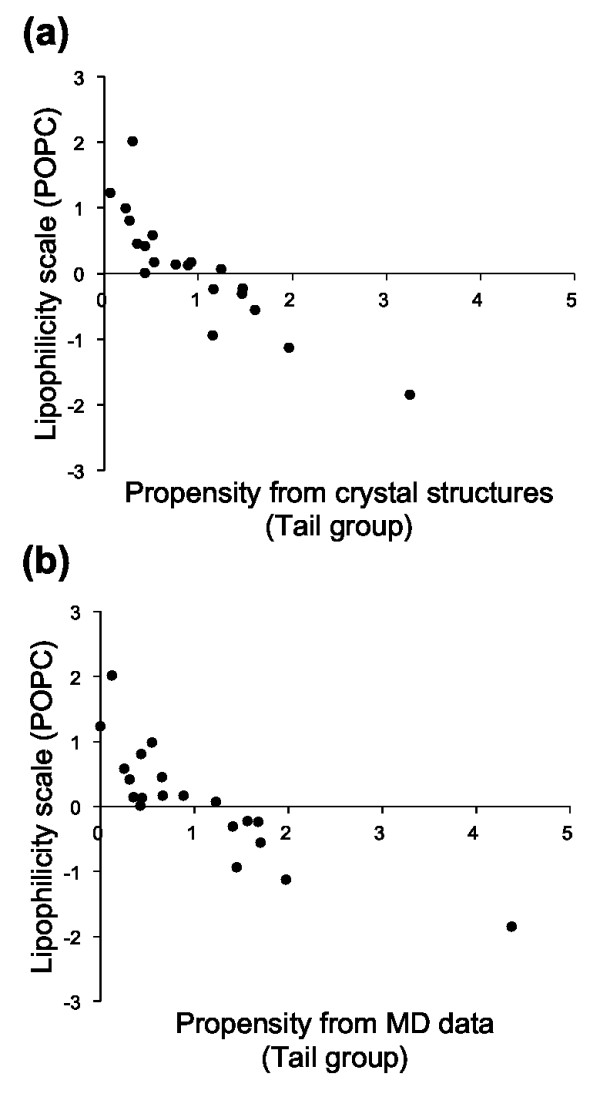
**Scatterplots of amino acid propensities for interacting with lipid tail groups versus the POPC lipophilicity scale**. Propensities for lipid tail groups derived from (a) crystal structures and (b) MD data. The correlation coefficients were -0.87 and -0.84 for the propensities from crystal structures and MD data, respectively.

The propensities for the tail group atomic contacts, derived from both the crystal structure and MD datasets, were highly correlated with the lipophilicity scales (with the correlation ranging from 0.75 to 0.87, Figure 2). However, the propensities for the head group atomic contacts were poorly correlated with the lipophilicity scales (with the correlation ranging from 0.06 to 0.28). This observation suggests that the lipid tail group propensities can be largely described by the free energy of transfer of model peptides.

### Comparison with non-TM data

Amino acid propensities for contacting with lipids were derived also from a set of non-TM proteins and compared with those derived from the TM dataset. A summary of the Chi-square statistics for lipid contacts of all 20 amino acid residues in the TM and non-TM proteins is presented in Table 5.

**Table 5:** Lipid contact statistics in TM and non-TM proteins with (a) head group and (b) tail group atoms

	Transmembrane (TM) proteins					Non-transmembrane (non-TM) proteins				
		
	Obs^a^	Exp^a^	Counts	Signed chi-square	P-value	Obs^a^	Exp^a^	Counts	Signed chi-square	P-value
**(a) Head group**										
TRP^†, ‡^	**22**	**9.12**	**290**	**18.19**	**2.00E-05**	3	2.23	84	0.27	6.06E-01
PHE^†, ‡^	**35**	**22.65**	**720**	**6.74**	**9.43E-03**	13	9.06	341	1.72	1.90E-01
TYR^†, ‡^	**28**	**12.90**	**410**	**17.69**	**2.60E-05**	**21**	**7.25**	**273**	**26.08**	**3.27E-07**
LEU	28	37.40	1189	-2.36	1.24E-01	20	21.85	823	-0.16	6.92E-01
ILE	**11**	**23.18**	**737**	**-6.40**	**1.14E-02**	9	11.58	436	-0.57	4.49E-01
CYS	2	4.25	135	-1.19	2.76E-01	2	4.51	170	-1.40	2.37E-01
MET	11	11.51	366	-0.02	8.80E-01	1	4.22	159	-2.46	1.17E-01
GLY^‡^	**15**	**28.06**	**892**	**-6.07**	**1.37E-02**	15	13.54	510	0.16	6.92E-01
VAL	**16**	**26.70**	**849**	**-4.29**	**3.83E-02**	14	15.11	569	-0.08	7.75E-01
SER	14	20.57	654	-2.10	1.47E-01	13	15.61	588	-0.44	5.08E-01
THR	16	19.53	621	-0.64	4.24E-01	5	10.62	400	-2.98	8.45E-02
ALA	**16**	**29.85**	**949**	**-6.42**	**1.13E-02**	12	17.55	661	-1.76	1.85E-01
HIS^†, ‡^	**16**	**8.05**	**256**	**7.85**	**5.09E-03**	7	5.47	206	0.43	5.13E-01
ASN^†, ‡^	**22**	**11.45**	**364**	**9.73**	**1.82E-03**	11	9.00	339	0.44	5.06E-01
PRO	9	15.98	508	-3.05	8.09E-02	6	10.36	390	-1.83	1.76E-01
GLN^†, ‡^	**14**	**8.30**	**264**	**3.91**	**4.80E-02**	12	9.77	368	0.51	4.76E-01
ARG^†, ‡^	**32**	**13.24**	**421**	**26.58**	**2.53E-07**	**20**	**10.41**	**392**	**8.84**	**2.95E-03**
LYS^†, ‡^	**20**	**12.20**	**388**	**4.98**	**2.56E-02**	20	13.01	490	3.75	5.27E-02
ASP	6	11.73	373	-2.80	9.43E-02	**5**	**11.79**	**444**	**-3.91**	**4.80E-02**
GLU	8	14.34	456	-2.80	9.40E-02	7	13.04	491	-2.80	9.45E-02
**(b) Tail group**										
TRP^†, ‡^	**42**	**12.9**	**290**	**65.46**	**5.93E-16**	**9**	**3.07**	**84**	**11.48**	**7.05E-04**
PHE^†, ‡^	**63**	**32.1**	**720**	**29.82**	**4.75E-08**	**35**	**12.45**	**341**	**40.84**	**1.66E-10**
TYR^†, ‡^	21	18.3	410	0.41	5.22E-01	**20**	**9.97**	**273**	**10.10**	**1.49E-03**
LEU^†, ‡^	**85**	**53.0**	**1189**	**19.37**	**1.08E-05**	**68**	**30.05**	**823**	**47.92**	**4.43E-12**
ILE^†, ‡^	**48**	**32.8**	**737**	**7.01**	**8.12E-03**	**33**	**15.92**	**436**	**18.33**	**1.86E-05**
CYS^†^	7	6.0	135	0.16	6.88E-01	5	6.21	170	-0.23	6.28E-01
MET^†, ‡^	24	16.3	366	3.63	5.67E-02	**13**	**5.81**	**159**	**8.92**	**2.83E-03**
GLY	**17**	**39.7**	**892**	**-13.01**	**3.10E-04**	**4**	**18.62**	**510**	**-11.48**	**7.03E-04**
VAL^†, ‡^	47	37.8	849	2.23	1.36E-01	**34**	**20.78**	**569**	**8.42**	**3.72E-03**
SER	26	29.1	654	-0.34	5.61E-01	**8**	**21.47**	**588**	**-8.45**	**3.65E-03**
THR	21	27.7	621	-1.61	2.05E-01	**7**	**14.61**	**400**	**-3.96**	**4.66E-02**
ALA	39	42.3	949	-0.25	6.14E-01	24	24.14	661	0.00	9.78E-01
HIS	6	11.4	256	-2.56	1.10E-01	3	7.52	206	-2.72	9.92E-02
ASN	**7**	**16.2**	**364**	**-5.24**	**2.21E-02**	**3**	**12.38**	**339**	**-7.11**	**7.69E-03**
PRO	**8**	**22.6**	**508**	**-9.46**	**2.10E-03**	9	14.24	390	-1.93	1.65E-01
GLN	6	11.8	264	-2.82	9.30E-02	**2**	**13.44**	**368**	**-9.73**	**1.81E-03**
ARG	**5**	**18.8**	**421**	**-10.09**	**1.49E-03**	7	14.31	392	-3.74	5.32E-02
LYS	**4**	**17.3**	**388**	**-10.21**	**1.40E-03**	**8**	**17.89**	**490**	**-5.47**	**1.94E-02**
ASP	**1**	**16.6**	**373**	**-14.68**	**1.28E-04**	**2**	**16.21**	**444**	**-12.46**	**4.16E-04**
GLU	**6**	**20.3**	**456**	**-10.09**	**1.49E-03**	**3**	**17.93**	**491**	**-12.43**	**4.22E-04**

Statistically significant values (p-value <0.05 or 95% significance) are in bold font. The amino acids are sorted in the ascending order of the lipophilicity scale for POPC interface.

^†, ‡ ^The dagger and double-dagger symbols are used to show residues in which observed contacts are more than expected for TM and non-TM proteins, respectively.

^a ^Obs and Exp stand for observed and expected number of counts, respectively.

Despite some small differences in the degree of preference (e.g., ASN contacts with lipid head groups being statistically significant only in the TM dataset), no amino acids were exclusively preferred in either dataset. Out of the 40 comparisons in Table 5 (for 20 amino acids in each type of contacts), only two occurrences were found such that the number of observed contacts was higher than expected in TM and lower than expected in non-TM or vice versa (GLY for the head group contacts and CYS for the tail group contacts).

To summarize, we found that an almost identical set of amino acids were used to form lipid contacts in the TM and non-TM proteins, with only small differences in the statistical significance of over- or under-representation.

## Discussion

We showed that the patterns of membrane protein-lipid interactions obtained from both the crystal structures and MD trajectories were highly correlated with each other (Figure 1). We also showed that the recognition of lipid tail groups by amino acid residues can be described by the lipophilicity scales (Table 4) and had the same tendency with non-TM proteins (Table 5), while lipid head groups demonstrated considerable variation among individual proteins. We discuss here how our observations can be associated with existing experimental data and previously proposed concepts concerning protein-lipid interactions. We also elaborate on the high propensities of TRP residue for the membrane protein-lipid interface.

### Relation of Amino acid propensities to lipid-membrane protein interaction

Since membrane proteins are generally crystallized with detergent molecules used for solubilization and purification, the lipid molecules that remain in the crystal are considered those that are tightly bound to the membrane proteins. On the other hand, the lipid molecules in the first shell, also known as the annular shell around a membrane protein, are in direct contact with the protein and form weak and non-specific interactions according to spin-label EPR and fluorescence quenching experiments [40,41]. Thus, intuitively, the amino acid propensities from the crystal structures should correspond to propensities for interacting with tightly-bound lipid molecules, while those from the MD trajectories should correspond to propensities for interacting weakly with lipid molecules in the annular shell (although some of these lipid molecules can be tightly bound). It is, therefore, non-trivial that we have observed such a high level of correlation between the propensities derived from these two datasets (Figure 1). Assuming that the tight binding of lipids is achieved by forming a special binding pocket on the surface of a protein, the amino acid composition of such binding pockets appears to be no different from that of other surface positions. This result implies that no special chemical interaction is required for achieving the tight binding of at least the tail portion of lipid molecules, but transmembrane helix packing may create a specific binding pocket for specific lipid types for the protein's function.

Experimental studies of the potassium channel KcsA [4,42] suggest that the tightly-bound lipids can be essential for its stability and function. The amino acid residues that interact with these tightly-bound lipids must have been selected during the course of evolution. However, our results suggest that these amino acids have been selected not necessarily based on their ability to form special chemical interactions with lipid tails but rather, they are general lipid-binding surface amino acids and happened to have been utilized for offering a physical basis of strong interaction.

For the head group contacts, although the TM and non-TM datasets produced a similar trend (Table 5), a weaker correlation was observed between the propensities derived from the crystal structure and MD datasets than that for the tail group contacts (Figure 1). The difference between the head and tail contacts may be attributable to the larger standard error for the propensities for the head contacts (Table 3). The propensity values were computed for each protein and then averaged and thus, the larger standard error indicates a larger variance among the propensity values derived from different proteins. Indeed, a variety of modes of interaction have been observed between the protein and lipid head groups in our dataset. Head groups of lipids often show disorder in high-resolution X-ray structures even when their tail groups are observed [40,43]. In our dataset, the head groups of tightly-bound lipids were completely or mostly disordered in rhodopsin (1gzm_A), sensory rhodopsin (1xio_A), succinate:ubiquinone oxidoreductase SQR (2h89_C) and halorhodopsin (3a7k_A); and fully or partially observed but not forming any hydrogen bond in bacteriorhodopsin (1x0i_1), SQR (1zoy_D), V-Type Na^+^-ATPase (2bl2_I) and ligand-gated ion channel GLIC (3eam_C). In other cases, the head groups appeared and formed hydrogen bonds, while the tail groups were disordered in Ca^2+^-ATPase (2eau_A), rhomboid protease GlpG (2irv_B), potassium channel Kir (2wll_D) and nitrate reductase A NarGHI (3egw_C).

Experimental studies have shown that differences in the chemical composition of the lipid head group affect the stability and function of membrane proteins, including KcsA, MscL, Ca^2+^-ATPase and others. Considering all these observations, the role of lipid head-protein interactions is likely to vary among different types of membrane proteins and this notion is consistent with the head contact propensities obtained in this paper, which were diverse and more complex than the tail contact propensities.

### Concentration of TRP at a lipid-water interface for anchoring the protein to the membrane

In both the crystal structure and MD datasets, we observed a conspicuously high propensity of TRP residues for contacting lipid molecules (Figure 1), indicating that TRP favors positions in a membrane protein that allow interaction with lipids.

Although TRP is generally not an abundant residue, either in membrane or soluble proteins [16], TRP has been reported to occur frequently near the membrane boundaries [44-46], as confirmed by our recent statistical analysis [16]. Systematic experimental studies using model peptides and proteins have also produced a similar picture [47-50]. (See Killian and von Hejine [51] for a review and examples of high-resolution structures are found in Lee [40].)

The amphiphilic nature of TRP (and also TYR) residues explains why TRP favors to locate at a water-lipid interface; these amphiphilic residues are thought to be locking the membrane protein into the correct location and orientation like anchors or floats at the membrane-water interface. Sansom and colleagues have observed the interfacial anchoring behavior of the amphiphilic residues in their MD simulations of both the outer membrane protein OmpA and the potassium channel KcsA [18].

All indications are that the significantly high propensities in Figure 1 were obtained as a consequence of the combined effect of the general low abundance and the amphiphilic nature of TRP.

## Conclusions

We analyzed lipid preferences of membrane proteins at atomic resolution, which were divided into those for lipid head and tail groups, by using a combination of data from crystal structures and MD simulations. The results revealed a common pattern of lipid tail-amino acid interactions in both datasets, suggesting that tightly-bound lipid molecules and lipids in the annular shell interact with membrane proteins in a similar manner, largely explained by general lipophilicity. On the other hand, lipid head-amino acid interactions showed a more complicated and variable pattern and are likely to affect the specific function of individual proteins. We also showed that TM and non-TM proteins utilize essentially an identical set of amino acids for interacting with lipid head and tail groups.

## Authors' contributions

MM and AVSKMK created the dataset, carried out analysis and drafted the manuscript. SA performed the statistical analysis. TM and YS carried out the molecular dynamics. KM conceived of the study, and participated in its design and coordination and helped to draft the manuscript. All authors read and approved the final manuscript.

## Supplementary Material

Additional file 1 **This file includes Figure S1**. Click here for file
