# Supplementary material for: Lipid recognition propensities of amino acids in membrane proteins from atomic resolution data
Source: BMC Biophys. 2011 Dec 14;4:21. doi: 10.1186/2046-1682-4-21 (PMC3747235; doi:10.1186/2046-1682-4-21)
Supplement: Additional file 1 — This file includes Figure S1. [file 2046-1682-4-21-S1.PDF]

Supplementary Information

Lipid recognition propensities of amino acids in membrane proteins from atomic resolution data

Mizuki Morita, A. V. S. K. Mohan Katta, Shandar Ahmad, Takaharu Mori, Yuji Sugita, Kenji Mizuguch

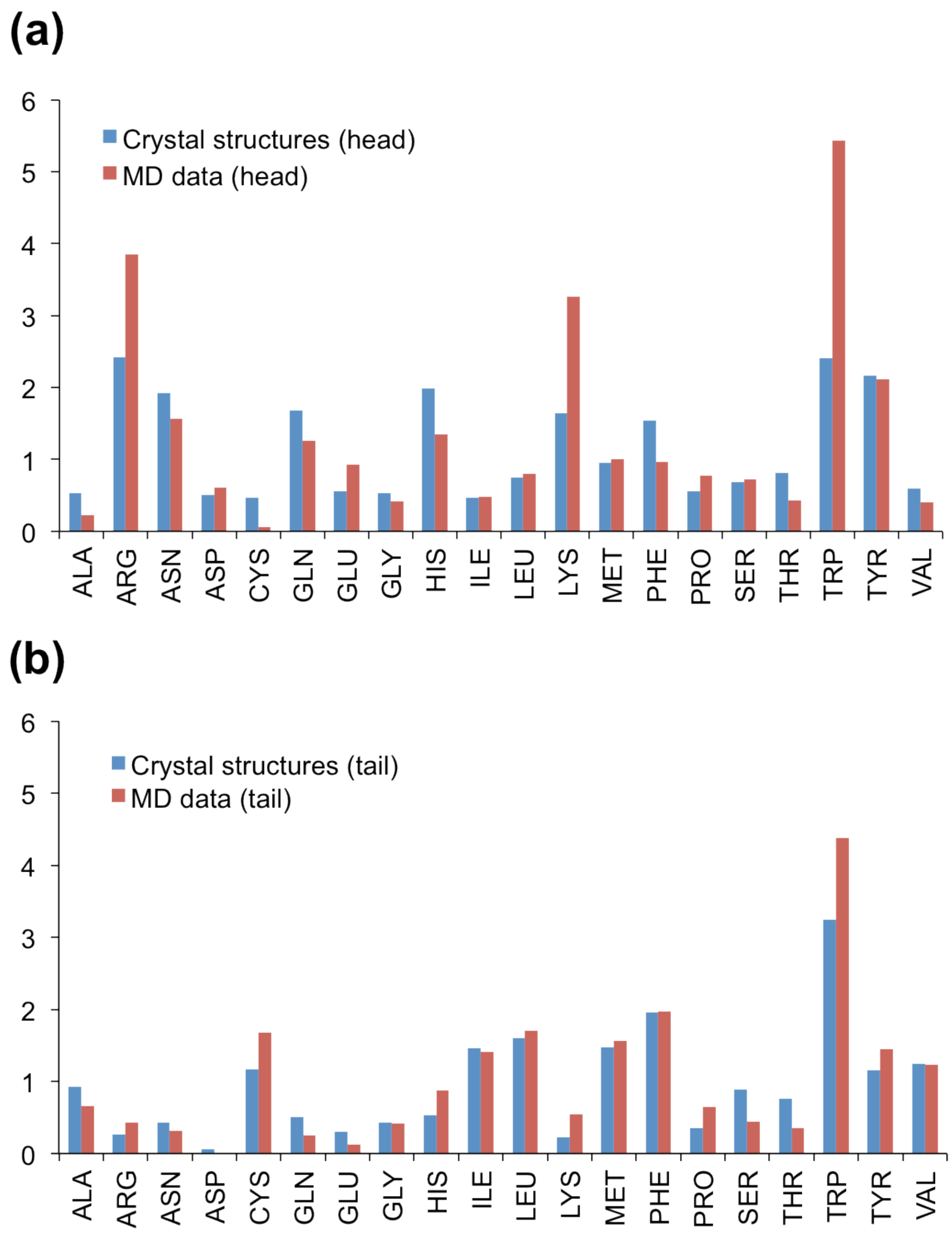

Figure S1 - Bar graphs of propensities of each amino acid for interacting with lipid head and tail groups. For (a) lipid head and (b) tail groups. Blue bars represent propensities derived from crystal structures and red bars from MD data.
